# User Localization in Complex Environments by Multimodal Combination of GPS, WiFi, RFID, and Pedometer Technologies

**DOI:** 10.1155/2014/814538

**Published:** 2014-03-31

**Authors:** Trung-Kien Dao, Hung-Long Nguyen, Thanh-Thuy Pham, Eric Castelli, Viet-Tung Nguyen, Dinh-Van Nguyen

**Affiliations:** MICA Institute (HUST-CNRS/UMI 2954-INP Grenoble), Hanoi University of Science and Technology, Hanoi, Vietnam

## Abstract

Many user localization technologies and methods have been proposed for either indoor or outdoor environments. However, each technology has its own drawbacks. Recently, many researches and designs have been proposed to build a combination of multiple localization technologies system which can provide higher precision results and solve the limitation in each localization technology alone. In this paper, a conceptual design of a general localization platform using combination of multiple localization technologies is introduced. The combination is realized by dividing spaces into grid points. To demonstrate this platform, a system with GPS, RFID, WiFi, and pedometer technologies is established. Experiment results show that the accuracy and availability are improved in comparison with each technology individually.

## 1. Introduction

For the last few years, the definition of intelligent environments has been introduced [1] and many researches have been following this direction. One of the top priority researches considers location-based services (LBS), in which localization technologies play an indispensable role. Many user localization technologies and methods have been proposed for either indoor or outdoor environments. Under specified circumstances and requirements from applications, independent technologies were proposed and optimized [2, 3]. Among the most used technologies are GPS, RFID, other methods based on WiFi, camera, accelerometer, microphones, and so forth [4].

However, building a localization platform for large and complex areas like campus or city, which includes both outdoor and indoor zones, is still a problem as each localization technology has its own drawbacks. For example, GPS is not good with indoor environments when the number of visible satellites is reduced because of walls; WiFi positioning is only suitable for user localization with low precision because of its accuracy varying from few meters to tens of meters; RFID, a proximity scheme, is limited in a small range since RFID readers cannot be installed at every location.

In order to overcome the limitations in each technology and provide better results in both precision and availability characteristics, a remarkable number of researches combining multiple localization technologies have been proposed. Pfeifer [5] introduced a design to extract results from localization technologies as useful information in real time. However, it lacked ideas and algorithms about how those results should be fused and analysed to produce better results. Yeh et al. [6] combined GPS, WiFi, and Zigbee into a system which can notably improve localization results in indoor areas. Martin [7] used WiFi and a pedometer in smartphones for high-precision localization applications. Other few hybrid systems showed improvements in precision, but these systems depended on specific technologies and lacked availability characteristics, such as RFID, WiFi, and camera [8]. None of those researches proposed a general approach or fusing algorithm to combine highly heterogeneous technologies.

In this paper, a general approach to multimodal localization system which combines multiple technologies based on the idea of dividing spaces into grid points is proposed. The system does not depend on any specific localization technologies but is built to be an open platform so that multiple heterogeneous localization technologies can be integrated into, since more technologies applied means we have more information to improve precision as well as availableness.

The remainder of this paper is organized as follows. In Section 2, the general architecture of the localization platform for integration of heterogeneous technologies is explained. After that, a system based on this platform with GPS, WiFi, RFID, and pedometer technologies is presented in Section 3. The experiment results and discussions are given in Section 4. Finally, concluding remarks are given in Section 5.

## 2. General Localization Platform

### 2.1. General Approach

To determine the user location, space is divided into grid points. At each point, the user appearance probability and precision are calculated with information provided from all available localization technologies. Besides, each application may require a different acceptable precision, so on the basis of the given precision from each application, the location with highest probability is chosen as user localization result:

(1) $$\begin{array}{c} x_{\text{res}}=x\;\text{if}\;\;\text{Pr}\left(x\right)=\overset{n}{\underset{i=1}{\max}}\left\{\text{Pr}\left(i\right)\right\}\wedge \xi \left(x\right)\leq \xi^{*}, \end{array}$$

where Pr(**x**) and *ξ*(**x**) are probability and precision at location **x**, ξ* is the acceptable precision given by the application, *n* is the number of grid points, and **x**_res_ is the localization result.

Since each localization technology provides results at different moments, the time difference between the last result produced by a certain technology and the current system time can affect the final result. Therefore, the probability at point **x** is defined as

(2) $$\begin{array}{c} \text{Pr}\left(x\right)=\overset{k}{\underset{i=1}{\sum }}P_{i}\left(x\right)R_{i}\exp\left(-\lambda_{i}\Delta t_{i}\right), \end{array}$$

where *P*_*i*_(**x**) is probability at point **x**, *R*_*i*_ is reliability constant (i.e., RFID results are more reliable compared to those of GPS), *λ*_*i*_ is time decay constant of the* i*th technology, Δ*t*_*i*_ is the time difference between the last result and the current system time, and *k* is the number of localization technologies in use. The precision at point **x** is defined as

(3) $$\begin{array}{c} \xi \left(x\right)=\overset{k}{\underset{i=1}{\min}}\left\{\xi_{i}\left(x\right)\right\}, \end{array}$$

where *ξ*_*i*_(**x**) is the precision of the* i*th technology at point **x**.

For localization technologies (such as GPS, RFID, and camera) providing results consisting of location and precision, *P*_*i*_(**x**) can be extracted from normal distribution using the empirical rule:

(4) $$\begin{array}{c} P_{i}\left(x\right)=\left(\frac{1}{\sigma_{i}\sqrt{2\pi }}\right)\exp\left(-\frac{d_{i}^{2}\left(x\right)}{2\sigma_{i}^{2}}\right), \end{array}$$

where *σ*_*i*_ = 3*ξ*_*i*_, *d*_*i*_(**x**) is the distance from point **x** to user location provided by the* i*th technology. For technologies based on user movement (such as accelerometer and pedometer), probability information *P*_*i*_(**x**) can be determined by combining the last localization result and normal distribution using (4). However, the value of *σ*_*i*_ is replaced by 3*ξ*_*i*_′, where *ξ*_*i*_′ is the last precision value of the* i*th technology at point **x**.

If the environment is large where the number of points is big, it is possible to reduce the searching time by first gridifying the space with fewer points to find a rough position and then repeating the same process once or twice with the subspace around this rough position for fine tuning.

**Figure 1:**
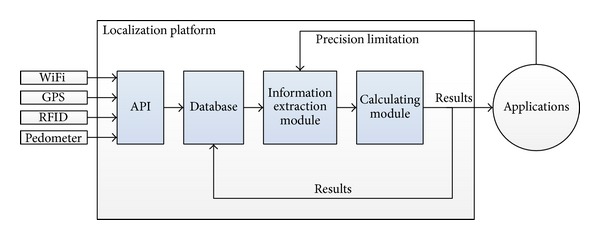
General architecture of localization platform.

The system, as illustrated in Figure 1, uses results from multiple technologies as inputs and gives out user location to applications as output. Event-driving approach is used; that is, each input will provide new localization results to ensure real-time availability. API module contributes as an open platform to extract useful information from inputs and store it in the database. Information extraction module extracts necessary information from database as well as receives the precision limitation from applications then provides them to the next module. Calculating module does the main processing function and produces user localization result. Depending on applications and their required precisions, results would be different. Afterwards, the outputs including user location, precision, and probability are sent to applications and fed back to be stored in database for next calculation tasks.

### 2.2. Parameter Optimization

In the localization system, for each integrated subjacent technology, the following parameters need to be determined: *R*_*i*_, *λ*_*i*_. Besides, each technology may have its own parameters. To this end, a genetic algorithm (GA) is used in this study to find the optimal parameter set. Genetic algorithms [9] are global search techniques modeled after the natural genetic mechanism to find approximate or exact solutions to optimization and search problems. In a GA, each parameter to be optimized is represented by a gene; moreover, each individual is characterized by a chromosome, which is actually the above set of parameters awaiting optimization. To assess the quality of an individual, a fitness function (objective function, or cost function) must be defined. For the localization module, the fitness function Φ is defined as the root mean square of the localization error

(5) $$\begin{array}{c} \Phi ={\left(\frac{1}{N}\overset{N}{\underset{i=1}{\sum }}{\left({\hat{x}}_{i}-x_{i}\right)}^{2}+{\left({\hat{y}}_{i}-y_{i}\right)}^{2}+{\left({\hat{z}}_{i}-z_{i}\right)}^{2}\right)}^{1/2}, \end{array}$$

where *N* is the number of measurements and $\left({\hat{x}}_{i},{\hat{y}}_{i},{\hat{z}}_{i}\right)$ and (*x*, *y*, *z*) are the estimated and the real user position, respectively.

**Figure 2:**
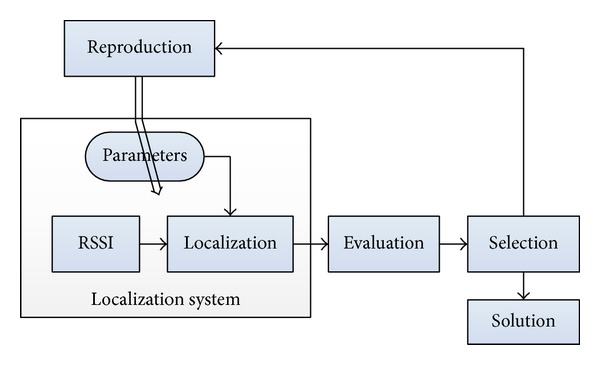
Optimization of system parameters using GAs.

In brief, a GA starts by generating an initial population (or initial generation); then, the quality of each individual is evaluated by using the fitness function. After one generation, only the advantageous individuals survive and reproduce to generate a new population. By this process of selection from generation to generation, the quality of the offspring is improved in comparison with their ancestors, as shown in Figure 2. During the creation of a new generation, a portion of the surviving individuals is recombined randomly via the so-called crossover and mutation operations, being adopted from natural evolution.

At the initial phase, the population consists of randomly generated heterogeneous chromosomes. After that, all chromosomes go through three principal parts: evaluation, selection, and reproduction modules. The population will be improved as fitter offspring individuals to replace parents. The procedure is repeated until either a maximum number of generations are reached or an optimal solution is obtained, whichever is earlier. In this study, the searching procedure is repeated until a parameter set satisfying the optimization criteria with acceptable tolerance is found.

The advantages of GAs over other searching algorithms are that they do not require any gradient information neither continuity assumption in searching for the best parameters and that they can explore many characteristics at once, which is necessary when dealing with complex problems.

## 3. System Implementation

The number of studies to monitor location using smartphones has increased recently due to their advantages and popularity: embedded GPS radio, WiFi functioning, and pedometer using built-in accelerometer sensor [10] as well as other sensors (Bluetooth, orientation, magnetic field, etc.). RFID tags are widely deployed in many applications with the advantages of their small size and variables in contents. We decided to first implement four technologies as inputs in the system: GPS, WiFi, pedometer from Android smartphones, and RFID positioning by RFID tags.

### 3.1. WiFi

With conventional receivers, distance to WiFi APs can be estimated from the measured RSSI with the help of a RF propagation model which is constructed based on the fact that a radio wave that travels through a certain environment will undergo specific types of signal attenuation. To start off, the empirical model widely used in previous works [11] is considered:

(6) $$\begin{array}{c} P=P_{0}-10n\log\left(\frac{r}{r_{0}}\right), \end{array}$$

where *P*_0_ is the known signal power at a reference distance *r*_0_ in dBm, *P* is the signal power at an unknown distance *r*, and *n* is the path-loss exponent.

**Figure 3:**
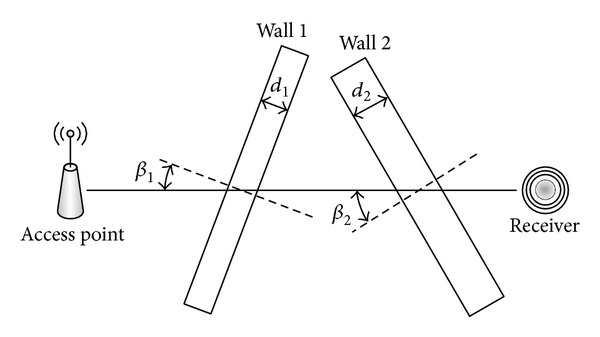
WiFi signal attenuation through walls/floors.

Equation (6) is a propagation model in an environment without obstacle between the AP and the receiver. When walls and floors are considered in calculation, attenuation due to these factors must be included, and the propagation equation becomes

(7) $$\begin{array}{c} P=P_{0}-10n\log\left(\frac{r}{r_{0}}\right)-k_{d}\overset{n_{w}}{\underset{i=1}{\sum }}\frac{d_{i}}{\cos\beta_{i}}, \end{array}$$

where *n*_*w*_ is the number of walls and floors in the middle, *d*_*i*_ and *β*_*i*_ are the thickness and angle of arrival to the* i*th wall/floor, respectively, and *k*_*d*_ is the attenuation factor per wall/floor thickness unit, as illustrated in Figure 3. In general cases, *k*_*d*_ can be extended to be dependent specifically on each wall/floor.

Equation (7) is a deterministic model; that is, the uncertainty of RSSI at a distance is not taken into account. In reality, given the RSSI *P*, the distance *r* might not be exactly the value calculated from (6) but is within a range around this value, which is denoted by $\overset{-}{r}$. To be more precise, $\overset{-}{r}$ will be the nominate value of the distance *r* with highest probability. To address this limitation, a probabilistic model is used, in which the distribution of the distance is assumed to follow the normal distribution with median $\overset{-}{r}$:

(8) $$\begin{array}{c} \rho \left(r,P\right)=\text{Pr}\left(r|P\right)=\left(\frac{1}{\sigma \sqrt{2\pi }}\right)\exp\left(-\frac{{\left(r-\overset{-}{r}\right)}^{2}}{2\sigma^{2}}\right), \end{array}$$

where $\sigma =k_{\sigma }\overset{-}{r}$ is the standard deviation, with *k*_*σ*_ being a constant.

From a tuple of RSSI information received from a WiFi receiving device, the system determines the location with maximal summation of probabilities corresponding to the visible APs, that is, maximizing *ρ*_Σ_(*x*, *y*, *z*) = ∑_*i*=1⋯*n*_AP__ *ρ*(*r*_*i*_(*x*, *y*, *z*), *P*_*i*_), where *n*_AP_ is the number of visible Aps; *ρ*_Σ_(*x*, *y*, *z*), the probability that the user is located at position (*x*, *y*, *z*); and *ρ*(*r*_*i*_(*x*, *y*, *z*), *P*_*i*_), the probability component based on the* i*th visible AP. The search process is achieved by gridifying the space surrounding the environment into a number of points and calculating *ρ*_Σ_ for each of them to find the point that maximizes *ρ*_Σ_.

### 3.2. GPS, RFID, and Pedometer

For GPS technology, we use the built-in GPS API from Android platform with extraction information including location (latitude, longitude, and altitude) and its precision. The pedometer is implemented using built-in sensors from Android smartphones. Using derivative applied to the smoothened signal from accelerometer, when the derivative is greater than threshold value, we detect a step [12, 13]. Direction of users is determined by geometry sensor with azimuth, roll, and pitch angle information. After a high precision localization from RFID, the pedometer can make a good localization in a short time.

With characteristics of RFID radio signal, it can be assured that at the moment user's RFID tag is recognized by a reader, the user is within 2 meters around it. The RFID reader position is stored in database and every time a RFID tag is read from a reader, information including user ID and RFID reader ID will be sent to system API. Afterwards user location will be calculated on the basis of the RFID reader location.

## 4. Experiment Results

**Figure 4:**
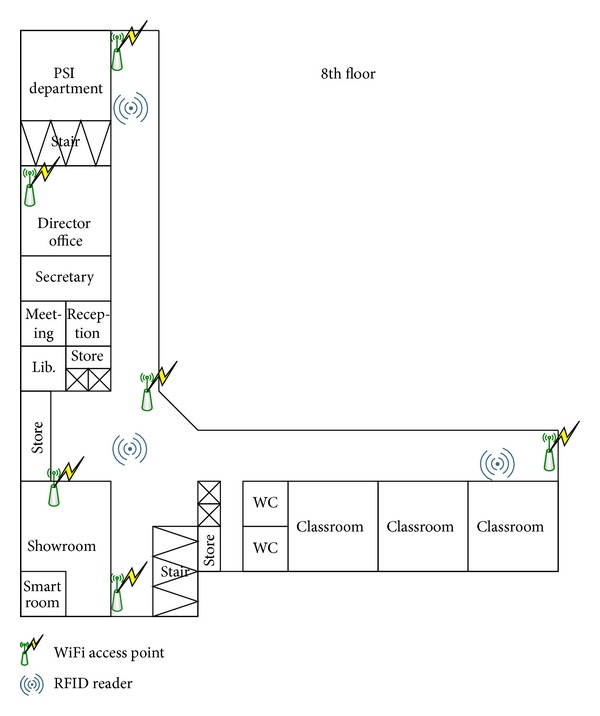
Testing environment.

Experimental scenario is taken in the 8th floor of an 11-floor building, as shown in Figure 4. A user holds a smartphone, walks starting from Point 1 straight to Point 2, Point 3, respectively, and then returns to Point 2 and Point 1 at the same path, with the total distance of 160 m. In this experiment, 3 RFID readers, 16 WiFi access points are set up for RFID and WiFi technologies.

First, data are collected to tune system parameters, as discussed in Section 2.2. A person holding the first smartphone moves slowly at a constant speed along the corridors on the three floors to measure the WiFi signal strengths. There are totally 672 measurements carried out for the training data. The training process using the GA is set up with configuration provided in Table 2. Using these data, the produced optimal parameter values are given in Table 3.

**Figure 5:**
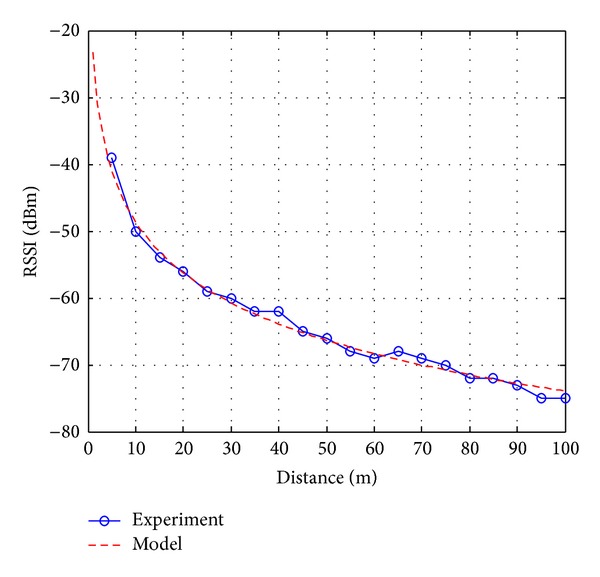
WiFi deterministic propagation model compared to measurements.

**Figure 6:**
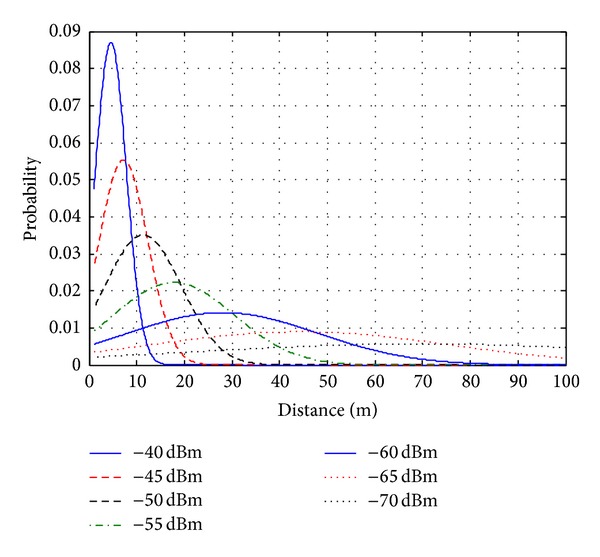
WiFi probabilistic propagation model.

With the optimized parameters, the WiFi propagation model in (6) is compared with measurements of RSSI to APs with no wall/floor in the middle of the lines of sight (LOS), as depicted in Figure 5. With a fixed AP, the smartphone is placed at distances from 5 to 100 m, with increment of 5 m. At each distance, 5 measurements are carried out, and then the average value is used and shown in this figure. It can be observed that the tuned model is closed to the experiment data, with highest error of 1.9 dBm. This comparison verifies the deterministic propagation model of WiFi signals.  Figure 6 shows the WiFi probabilistic propagation model in (7) with the tuned parameters. For each RSSI value, a probability distribution function (PDF) is established following the normal distribution with median value calculated with the help of the deterministic model.

**Figure 7:**
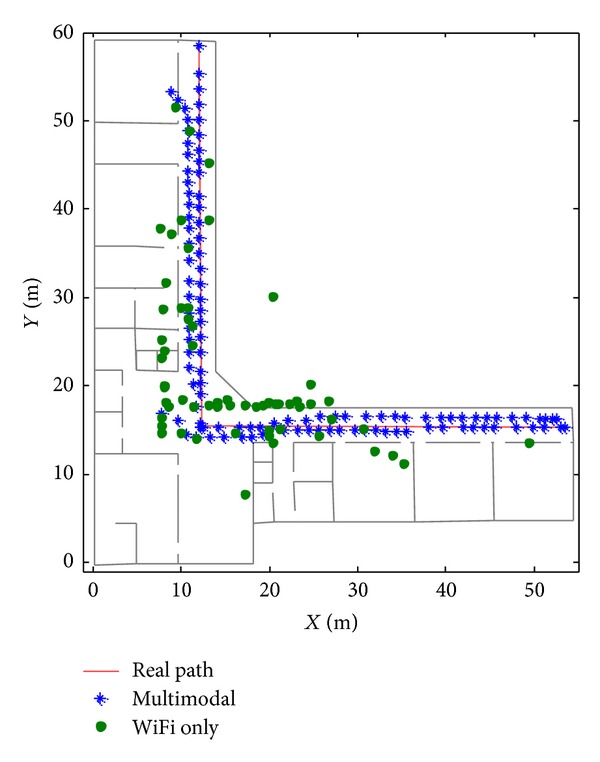
Localization results.

Results are compared and analysed between multimodal system and WiFi alone system.  Figure 7 shows user walking path and localization results in two cases using multimodal localization system, compared with the system using WiFi alone. It can be observed that multimodal-based results almost coincide with user walking path while WiFi-based results have big jumps and it is impossible to track position of user with only WiFi-based system.

**Figure 8:**
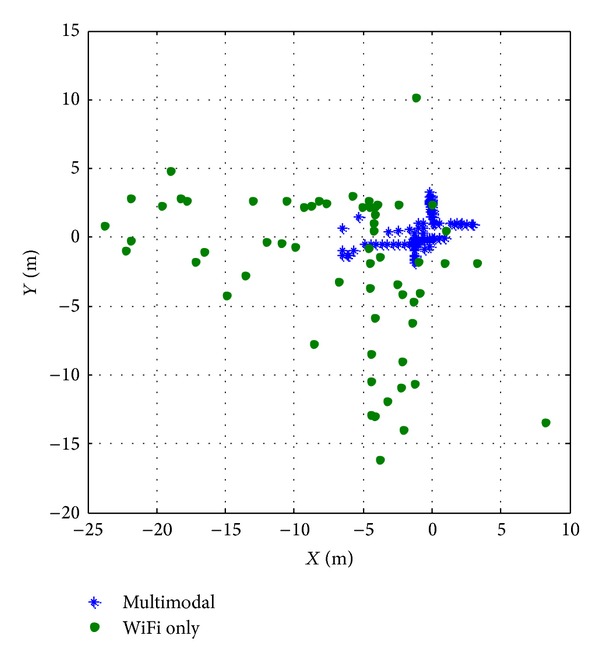
Localization error distribution.

**Figure 9:**
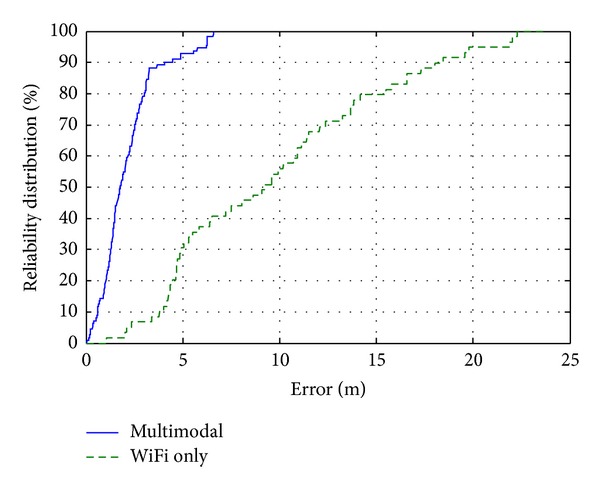
Localization reliability distribution.

Figures 8 and 9 present the error distribution and reliability distribution. While multimodal results gather in around 5 meters of error, WiFi results error distribution exceeds 20 meters. It is shown that current implementation for multimodal would be useful for applications with required precision of 5 meters. The error results are summarized in Table 1. Average error of multimodal system is about 4.5 times better, the maximal error is 3.6 times smaller, and the error at reliability of 90% is 4.5 times smaller compared to WiFi-based system. It can be seen that the multimodal system does a much better work, and localization results are improved significantly.

## 5. Conclusion

In this paper, an approach to build an indoor/outdoor multimodal system combining multiple localization technologies has been introduced. An implementation with WiFi, GPS, RFID, and pedometer technologies has been successfully built and demonstrated. Evaluation shows promising results for the approach in this system to continue in the future. For future works, the WiFi implementation algorithm will be improved. The pedometer implementation with smartphones also needs to be improved to work in pocket situation (currently, smartphones need to be held in hand). Third, it is necessary to think of some algorithms to turn on/off smartphone sensors on the basis of activities to save battery. Environment information constraints combining user's history records and Kalman filters could also be used to smoothen the output results. Next localization technology based on video processing will be implemented. At last, user's location privacy information sending between server and user security should be considered [14].

## Figures and Tables

**Table 1:** Multimodal and WiFi-only localization error comparison.

	Average error (m)	Maximal error (m)	Error at reliability of 90% (m)
Multimodal	2.18	6.62	4.07
WiFi only	9.99	23.84	18.44

**Table 2:** Genetic algorithm configuration.

Parameter	Value
Population size	20
Elite count	5
Crossover fraction	0.5
Time limit	No
Maximal generations	No
Tolerance	10^−6^
Selection	Uniform
Crossover	Scattered
Mutation	Uniform
Creation population	Uniform

**Table 3:** Optimized system parameters.

Technology	Reliability *R*_*i*_	Time decay constant *λ*_*i*_
WiFi	16.2	0.86
GPS	5.5	0.81
RFID	31.7	0.52
Pedometer	22.6	0.62
